# Autophagy of *Candida albicans* cells after the action of earthworm Venetin-1 nanoparticle with protease inhibitor activity

**DOI:** 10.1038/s41598-023-41281-4

**Published:** 2023-08-30

**Authors:** Sylwia Wójcik-Mieszawska, Kinga Lewtak, Ewa Skwarek, Dawid Dębowski, Agata Gitlin-Domagalska, Jakub Nowak, Jerzy Wydrych, Jarosław Pawelec, Marta J. Fiołka

**Affiliations:** 1 Department of Immunobiology, Institute of Biological Sciences, Faculty of Biology and Biotechnology, Maria Curie-Skłodowska University, Lublin, Poland; 2 Department of Cell Biology, Institute of Biological Sciences, Faculty of Biology and Biotechnology, Maria Curie-Skłodowska University, Lublin, Poland; 3 Department of Radiochemistry and Environmental Chemistry, Institute of Chemical Sciences, Faculty of Chemistry, Maria Curie-Skłodowska University, Lublin, Poland; 4 Department of Molecular Biochemistry, Faculty of Chemistry, University of Gdańsk, Gdańsk, Poland; 5 Malopolska Centre of Biotechnology, Jagiellonian University, Kraków, Poland; 6 Department of Functional Anatomy and Cytobiology, Institute of Biological Sciences, Maria Curie-Skłodowska University, Lublin, Poland

**Keywords:** Cell biology, Drug discovery, Microbiology

## Abstract

The present studies show the effect of the Venetin-1 protein-polysaccharide complex obtained from the coelomic fluid of the earthworm *Dendrobaena veneta* on *Candida albicans* cells. They are a continuation of research on the mechanisms of action, cellular targets, and modes of cell death. After the action of Venetin-1, a reduced survival rate of the yeast cells was noted. The cells were observed to be enlarged compared to the controls and deformed. In addition, an increase in the number of cells with clearly enlarged vacuoles was noted. The detected autophagy process was confirmed using differential interference contrast, fluorescence microscopy, and transmission electron microscopy. Autophagic vesicles were best visible after incubation of fungus cells with the Venetin-1 complex at a concentration of 50 and 100 µg mL^−1^. The changes in the vacuoles were accompanied by changes in the size of mitochondria, which is probably related to the previously documented oxidative stress. The aggregation properties of Venetin-1 were characterized. Based on the results of the zeta potential at the Venetin-1/KCl interface, the pHiep = 4 point was determined, i.e. the zeta potential becomes positive above pH = 4 and is negative below this value, which may affect the electrostatic interactions with other particles surrounding Venetin-1.

## Introduction

Long before the advent of modern Western medicine and the pharmaceutical industry, the animal kingdom was exploited in search of medicinal preparations. Earthworms were a source of food and medicinal substances in ancient cultures. Although the documented association of earthworms with medicine dates back to 1340 A.D.^1^, more specific scientific research was carried out only in the last decades. These invertebrates are an essential component of traditional Chinese medicine, in which preparations from earthworms are used to treat over 80 diseases, e.g. asthma, hypertension, ulcers, epilepsy, blood vessel diseases, or cancer. For a long time, earthworms have been widely used not only in China, Indonesia, or Japan but also in other countries of the Far East in the treatment of various chronic diseases^2^. In Burma and Laos, the earthworm body fluid is used to treat chickenpox^3^. In addition, in these countries, earthworms are baked, powdered, and eaten with coconut water to speed recovery. Decoctions of earthworms are known as a source of vitality for postpartum women. In India, infusions are used to reduce high fever and to treat digestive and nervous disorders. In Korea, it is widely believed that earthworms improve human health and prevent many diseases^4^. In Vietnam, a powder prepared from earthworms is an essential ingredient in many medicines used to treat bacterial and viral infections. In Iran, baked earthworms are added to bread to dissolve urinary stones. On the island of Java, there are special cultures of earthworms intended only for medical purposes. In South America, Ye'Kuan Indians use earthworms for consumption and to treat malaria and leukemia^5^.

The earthworm body contains many nutrients that are essential for human health. The most important among them are stearic and palmitic acids, unsaturated fatty acids, phosphatides, and cholesterol. These compounds are not only necessary but also effective in the treatment of various diseases^6^. The production of pharmacologically important compounds from earthworms is a new field of modern medicine.

Interactions between earthworms and microorganisms are still not fully understood. Since the living environment of earthworms is rich in fungal organisms, fungi are the main source of food for earthworms^7^ and at the same time, these invertebrates have mechanisms protecting them against pathogenic species. One of them is the action of the coelomic fluid (CF), which has been proven to kill fungal cells^8^. CF secreted by the dorsal pores contains many bioactive compounds, e.g. such enzymes as proteases^9–11^, lysozymes^12,13^, metalloenzymes^14^, and fibrinolytic enzymes^15^ as well as polysaccharides^16^, proteins^17–19^, and nutrients, etc. These factors have antibacterial^20,21^, antifungal^8,22^, anti-inflammatory^23^, antioxidant^23,24^, and anticancer^10,11,25–27^ effects.

Every year invasive fungal infections are diagnosed in 800 million patients worldwide, causing 1,660,000 deaths^28–30^, which is more than the death rate of *Escherichia coli* (950,000 deaths) or HIV/AIDS (864,000 deaths)^31^. The leading cause of serious fungal infections is *Candida albicans* accounting for 70% of total diseases caused by fungi, with mortality rates as high as 50%^30,32–34^. Systemic candidiasis caused by this yeast is found to be the second cause of death in premature babies^35^. *C. albicans* infections are also a serious problem in ICU units, where it is the first most common pathogen isolated in patients from Europe and the fourth in the US^36–38^.

*Candida albicans* owes its success in pathogenesis to a few factors. One of them is that this fungus is present as a commensal on human skin, mucosal membranes, and intestines^39–41^. Yeast proliferation is controlled by the immune system, but can escape immunity when the organism is weakened. The risk factors of candidemia development include antibiotic treatment, surgeries, chemotherapy, HIV infection, extremes of age, use of medical equipment, or prolonged hospital stay^39,42–44^. In favorable conditions, yeasts change their model of growth from unicellular to hyphae or pseudohyphae, form biofilm, and adhere to biotic and abiotic surfaces, which makes them hard to remove^39,41,43,45,46^. Biofilms are also very resistant against antibiotics, posing a serious threat to patients. Antibiotic treatment of candidiasis has it limits set by the toxicity of drugs, which cannot be administered to too weak patients. Another limitation is the increasing antibiotic resistance of fungi. Changes in the genetic material of *C. albicans* result in occurrence of multi-drug pumps or alterations in metabolic pathways, which makes antibiotics ineffective^39,47,48^.

The Venetin-1 nanoparticle- a protein-polysaccharide fraction (or complex) with antifungal activity against *C. albicans*, was isolated from CF of the earthworm *Dendrobaena veneta*. Its properties and chemical character were described in earlier publications^49–52^. The nanoparticle has been shown to have anticancer^25,53^, antiaggregating^54^, and immunostimulating properties^55^. Importantly, it does not show endotoxicity and cytotoxicity to normal human cells^49^. Venetin-1 has the ability to inhibit the 20S proteasome^56^. The aim of the present research was to carry out further characterization of the nanoparticle and the mechanism of its action on the cells of the fungus *C. albicans*.

## Materials and methods

### Earthworm breeding

**Figure 1:**
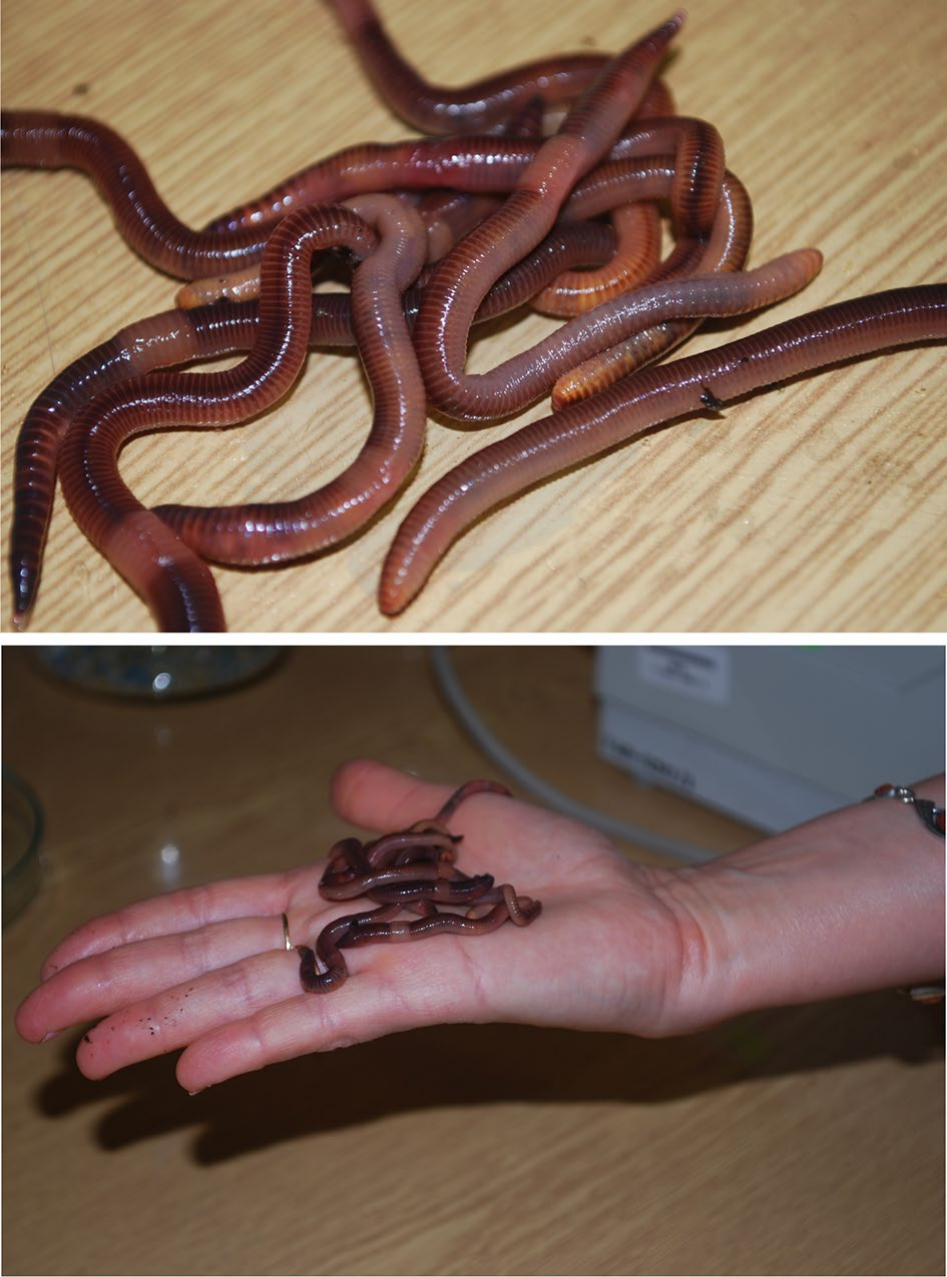
Mature *D. veneta* individuals.

Annelids representing the *D. veneta* species were kept in controlled conditions in the Department of Immunology, Maria Cure-Skłodowska University in Lublin. The earthworms were grown in 3 L plastic containers filled with compost soil in the dark at a temperature of 20 °C and a humidity of about 70–80%. The earthworms were fed twice a week with cooked vegetables. The diet was supplemented with pure cellulose needed for the for making cocoons. Adult specimens were selected for the experiments (Fig. 1).

### Extraction of Venetin-1 from earthworms

The earthworms were rinsed twice with distilled water and transferred to a plastic container filled with moist cellulose lignin for 24 h to cleanse their digestive tract. Next, a group of 10 earthworms was placed in a glass beaker, poured with 0.9% NaCl, and stimulated with an electrical current at 4.5 V for 30 s. CF obtained in this way was centrifuged for 10 min at 2400×*g* to separate the coelomocytes from the supernatant. The supernatant was then filtered through 0.22 µm Millipore filters and incubated at 70 °C for 10 min. Subsequently, the preparation was dialyzed in cellulose bags with a cut-off point of 12–14 kDa. Dialysis was carried out for 24 h at 4 °C. The resulting fluid was then transferred to Eppendorf tubes and lyophilized. The prepared substance was stored in a freezer (− 20 °C). For experimental purposes, the protein concentration was measured with the Bradford method^57^.

### Microorganisms

The clinical isolate of the *C. albicans* wild type strain, gifted by Prof. A. Kędzia from the Department of Oral Microbiology, Medical University of Gdańsk (Poland), and *Candida krusei* (*Issatchenkia orientalis*) ATCC 6258 (from the collection of the Department of Immunobiology, Maria Curie-Skłodowska University in Lublin, Poland) were used for the microbiological analysis.

### Preparation of C. albicans cell culture treated with Venetin-1

The *C. albicans* cell culture was maintained on solid Sabouraud’s medium. For the experiment, yeast cells were grown in liquid Sabouraud’s medium for 24 h at 28 °C. 20 µL of the yeast cell culture (10^7^ CFU) in “poor” YPD medium^51^ was incubated with 30 µL of streptomycin sulfate (Sigma) (water solution with concentration 1.4 mg mL^−1^), and Venetin-1 at the concentration of 25 µg mL^−1^, 50 µg mL^−1^, and 100 µg mL^−1^. The final volume of the cell cultures was 250 µL. The control culture was prepared without addition of Venetin-1. The cell cultures were incubated at 37 °C for 48 h with constant shaking.

### Differential interference contrast (DIC)

Yeast cells from the control culture and cells after incubation with Venetin-1 were transferred onto a microscopic slide in a volume of 2 µL, covered with a cover slip, and observed at magnification 60 × with the use of an Olympus BX61 optical microscope (Olympus, Japan) with DIC settings. The cells were measured using the ImageJ program (National Institute of Health, USA). 200 cells in each concentration were measured.

### Scanning electron microscopy (SEM)

The yeast cultures were centrifuged and the supernatant was suspended in a fixative pH = 7 (phosphate buffer, glutaraldehyde, saccharose) and incubated for 2 h at room temperature. Then, the fixative was discarded and 0.1 M phosphate buffer was added. Next, 1.5% OsO_4_ was added to the pelleted cells and centrifuged for 30 min at 2500×*g*. Then, OsO_4_ was removed and the cells were resuspended in 0.1 M phosphate buffer and centrifuged for 30 min at 2500×*g*. After that, the cells were dehydrated in acetone solutions with increasing concentrations: at 30%, 50%, 70%, and twice at 100%. The cell cultures were transferred onto SEM stages, stored in a desiccator with silica gel for 24 h, and sputtered with gold (K550X sputter coater, Quorum Technologies). The cells were imaged with a Vega 3 scanning electron microscope (Tescan, Czech Republic)^49^.

### Cryo-scanning electron microscopy (Cryo-SEM)

Samples with control *C. albicans* cells and cells after incubation with Venetin-1 were centrifuged and the supernatant was discarded. The pellet was suspended in 200 µL of a GH solution, centrifuged (10 min, 6000×*g*), and almost all of the supernatant was withdrawn. The fungal cells in the GH solution were placed in a sublimation chamber for 12 min at – 92 °C. Then, the samples were cut with a special blade in the preparation chamber and observed with the use of an EM ZEISS Ultra Plus SEM microscope (Carl Zeiss, Germany) at 5 kV.

### Fluorescence microscopy

*Candida albicans* cells for staining with fluorochromes were prepared as described previously in "Microorganisms". Cell suspensions were then incubated with different fluorochromes in proper conditions in order to visualize cell organelles. Stained yeast cells were observed using a confocal microscope (Carl Zeiss, Germany) with immersion.

#### Quinacrine dihydrochloride

Quinacrine dihydrochloride (Sigma-Aldrich) stains acidic organelles like autophagic bodies (yellowish fluorescence) and autophagosomes (blue fluorescence). Fungal cells for Quinacrine dihydrochloride staining were mixed in a proportion of 10 µL of the cell suspension with 10 µL of fluorochrome (1 mg mL^−1^ water solution) and incubated for 10 min at 37 °C in the dark. 2 µL of cell suspension was transferred onto glass slide, covered with a cover slip, and observed at a 436-nm excitation wavelength for Quinacrine.

#### Yeast LIVE/DEAD test

The LIVE/DEAD test is used to distinguish between metabolically active, inactive, and dead cells. However, such cellular organelles as mitochondria are stained as well. For the experiment, *C. krusei* cells were prepared in the same way as the *C. albicans* cells. The yeast cell suspensions were centrifuged, the supernatant was removed, and the pellets were suspended in GH buffer. Next, the yeast cell cultures were mixed with 10% FUN-1 in GH buffer in a 1:1 ratio and incubated for 30 min at 30 °C. 2 µL of stained cells were transferred onto microscopic slides and fluorescence was observed at a 480-nm emission wavelength^49^. Cells with red inserts in the cytoplasm were considered active, and green and yellow cells were inactive. Mitochondria fluoresced yellow-green at the periphery of the cells.

#### Acridine Orange

Acridine Orange (AO) is a fluorochrome indicating affinity for nucleic acids and acidic cellular compartments. An AO water solution (0.1 mg mL^−1^) was mixed with the yeast cell suspension in a 1:1 ratio and incubated for 10 min in the dark at room temperature. Samples in a volume of 2 µL were placed on a cover slip and observed at the excitation wavelength λ = 502 nm and emission wavelength λ = 526nm using a Zeiss/LEO 912AB microscope at 1000× magnification^51^. The cells undergoing the autophagy process were counted after acridine orange staining with the ImageJ program using the multitool. Approximately 500 cells in each sample were counted, and the experiment was repeated three times.

### Transmission electron microscopy (TEM)

Yeast cell cultures (preparation described in "Microorganisms") were fixed in GA (4% glutaraldehyde in 0.1 M cacodylate buffer pH = 7.2). Next, the *C. albicans* cultures were centrifuged (2500×*g*, 12 min) and rinsed twice with 0.1 M cacodylate buffer with centrifugation (2500×*g*, 12 min). Then, the pellet was fixed in 1.5% KMnO_4_ for 1 h 15 min at 5 °C. After this time, the cells were rinsed several times with distilled water until discolored. Embedded fungal cells were then contrasted with uranium acetate 1%, dehydrated with subsequent concentrations of ethanol, infiltered, and embedded in resin (LR White). The resin blocks were then cut into ultrathin sections and observed using a transmission electron microscope JEM-1400Flash (JEOL, Japan).

### Flow cytometry analysis

The flow cytometry analysis of live/dead yeast cells was performed using a mixture of Propidium iodide (10 µg mL^−1^ water solution) and Hoechst 33342 (5 µg mL^−1^ water solution) mixed in a proportion of 2:1. The cell cultures were incubated with a staining mixture at room temperature for 20 min in the dark; then, distilled water was added to a final volume of 800 µL^58^. The analysis was performed using a Guava easyCyte Flow Cytometer (Luminex, USA) with a 695/50 nm laser (Red-B-HLog), a 785/70 nm laser (NIR-R-HLog), Forward Scatter (FSC-HLog), Side Scatter (SSC-HLog), Threshold set on FSC at 68, and the total number of counted cells 5000. The obtained dot-plots were gated with the Quad Stat Marker for viable (upper left square) and dead (lower left square) cell regions.

### Cryo-TEM analysis of Venetin-1

Venetin-1 at a concentration of 1 mg mL^−1^ was ultrasonicated for 10 min at 40 °C in an ultrasonic chamber (Pol-Sonic, Poland). Then, the sample was vitrificated in a water solution on the TEM grid covered with Quantifoil R 2/2 carbon film (Quantifoil Micro Tools GmbH, Großlöbichau, Germany). Before observations, the grids were activated with oxygen plasma for 15 s in a Femto plasma cleaner (Diener Electronic, Germany). Next, 3 μL of the Venetin-1 suspension was transferred onto the grid, blotted with filter paper and immersed in liquid ethane for instant freezing by Vitrobot Mark IV (FEI Company, USA). Before observations, the samples were stored in liquid nitrogen. To transfer the specimens to the TEM microscope, they were loaded into the Gatan 626 Cryo-TEM holder (Gatan Inc., USA)^59^. The samples were observed using a Tecnai F20 X TWIN microscope (FEI Company, USA) with 200-kV acceleration voltage of the emission gun. An Eagle 4k HS camera (FEI Company, USA) was used to record the images.

### Dynamic light scattering domain-specific characterization of Venetin-1

The DLS technique with the Prometheus Panta was used to characterize the Venetin-1 nanoparticle. It provides the highest quality data on the biophysical characteristics of the analyzed material. Venetin-1 was dissolved in water at a concentration of 1.3 mg mL^−1^. The samples were loaded into eight capillaries acting as replicas, where a parallel analysis allowed us to analyze the homogeneity of the nanoparticles and their aggregation abilities under different temperature conditions.

### Zeta potential determination

The electrophoretic mobility, conductivity, and zeta potential measurements of the NaCl solution with the concentration of 10^–4^, 10^–3^ M and 10^–3^ M KCl were carried out using the Zetasizer Nano ZS90 by Malvern. Smoluchowsky’s equation was applied due to the value of κa ~ 150. The measurements were performed at a 100 ppm solid concentration of Venetin-1. The compound was added to the solution and subjected to dispersion using the ultrasound probe Sonicator XL 2020 produced by Misonix. Then, the suspension was poured into 125 mL flasks, and pH was established to be in the range of 3–11 using 0.1 M HCl and NaOH solutions. Five measurements of electrophoretic mobility, conductivity, and zeta potential were made for each solution.

### Inhibitory potency against selected serine proteases

All measurements were performed using a Fluostar Omega microplate reader (BMG Labtech, Germany) and 96-well black or transparent plates (BRAND, Germany). Recombinant bovine trypsin was purchased from Sigma-Aldrich (Germany) and chymotrypsin was provided by Bachem (Germany). Recombinant human matriptase-1/ST14 catalytic domain was purchased from R&D Systems (USA). Recombinant human matriptase-2 was obtained from Enzo Life Sciences (Switzerland). Soluble human recombinant furin was kindly gifted by Dr Anna Kwiatkowska^60^ from the group of Prof. Robert Day (Université de Sherbrooke, Canada).

The following assay buffers were used: 50 mM Tris–Cl, pH 8.3 with 20 mM CaCl_2_, (trypsin and chymotrypsin), 50 mM Tris–HCl pH 8.3 with 150 mM NaCl and 0.01% Triton X 100 (matriptase-1 and matriptase-2), and 100 mM HEPES pH 7.5 with 1 mM CaCl_2_ and 1.8 mg mL^−1^ of bovine serum albumin (furin). The following chromogenic or fluorogenic substrates were used in the assays: N^α^-Benzoyl-d,l-arginine 4-nitroanilide hydrochloride (BAPNA, Bachem, Germany) in a final concentration of 4.60 mM for trypsin, Suc-Ala-Ala-Pro-Leu-pNA (Bachem, Germany) in a final concentration of 1.69 mM for α-chymotrypsin, Boc-Gln-Ala-Arg-AMC (Pepta Nova, Germany) in a final concentration of 5 µM for matriptase-1 (MT1) and matriptase-2 (MT2), and Pyr-Arg-Thr-Arg-AMC (Pepta Nova, Germany) in a final concentration of 20 µM for furin. The concentrations of the enzymes were as follows: 138 nM of trypsin, 141 nM of chymotrypsin, 1.25 ng mL of MT1, 1.51 U mL of MT2, and 1.1 nM of furin.

The concentration of the bovine trypsin stock solution was determined spectrophotometrically at 410 nm by titration with the chromogenic burst substrate 4-nitrophenyl 4-guanidinobenzoate (NPGB, Sigma-Aldrich, USA). Later, the standardized trypsin solution was used to titrate turkey ovomucoid third domain OMTKY-3 (Sigma-Aldrich, USA) used as a mutual inhibitor of trypsin and chymotrypsin) with BAPNA as a substrate. Then, OMTKY-3 was used to determine the concentration of the α-chymotrypsin stock solution in the presence of its substrate Suc-Ala-Ala-Pro-Leu-pNA. In the case of MT1 and MT2, the concentrations were calculated in accordance with the information provided by the suppliers.

The tested compound was added to the appropriate protease in different concentrations and incubated in assay buffer at 37 °C for 30 min. After this time, the solution of an appropriate substrate was added. Reactions were monitored for at least 35 min at 37 °C. The final volume in each well was 200 µL. Measurements were carried out using excitation and emission wavelengths of 380 nm and 450 nm (for the substrates of MT1, MT2, and furin), and absorbance for the trypsin and chymotrypsin substrates was monitored at 410 nm. Percentage inhibition of an enzyme was calculated relative to the control sample without the inhibitor. Determination of the inhibitory activities were done according to Gitlin et al.^61^ and Gitlin-Domagalska et al.^62^.

The IC_50_ values (inhibitor concentrations giving 50% inhibition of enzyme activity) were calculated from plots of enzyme activity (% of the control sample) versus the inhibitor concentration using a four-parameter fit model (GraFit 5.0.12 Erithacus Software Ltd.). The IC_50_ values were determined from triplicate measurements with at least ten different inhibitor concentrations.

### Statistical analysis

Statistical analyses for assessment of changes in the number of *C. albicans* cells with autophagic bodies and changes in the cell size after the treatment with Venetin-1 were performed with the Statistica program (Tibco Software Inc., USA; serial number: JPZ009K288211FAACD-Q). The Shapiro–Wilk test was used to check the type of data distribution, and the Levene test was employed to determine the homogeneity of variance. The post-hoc Tukey HSD test and one-way ANOVA were used to examine the level of significance of differences in the number of cells with autophagic bodies and changes in the cell size.

## Results

### Flow cytometry analysis of *C. albicans* cells after treatment with Venetin-1

**Figure 2:**
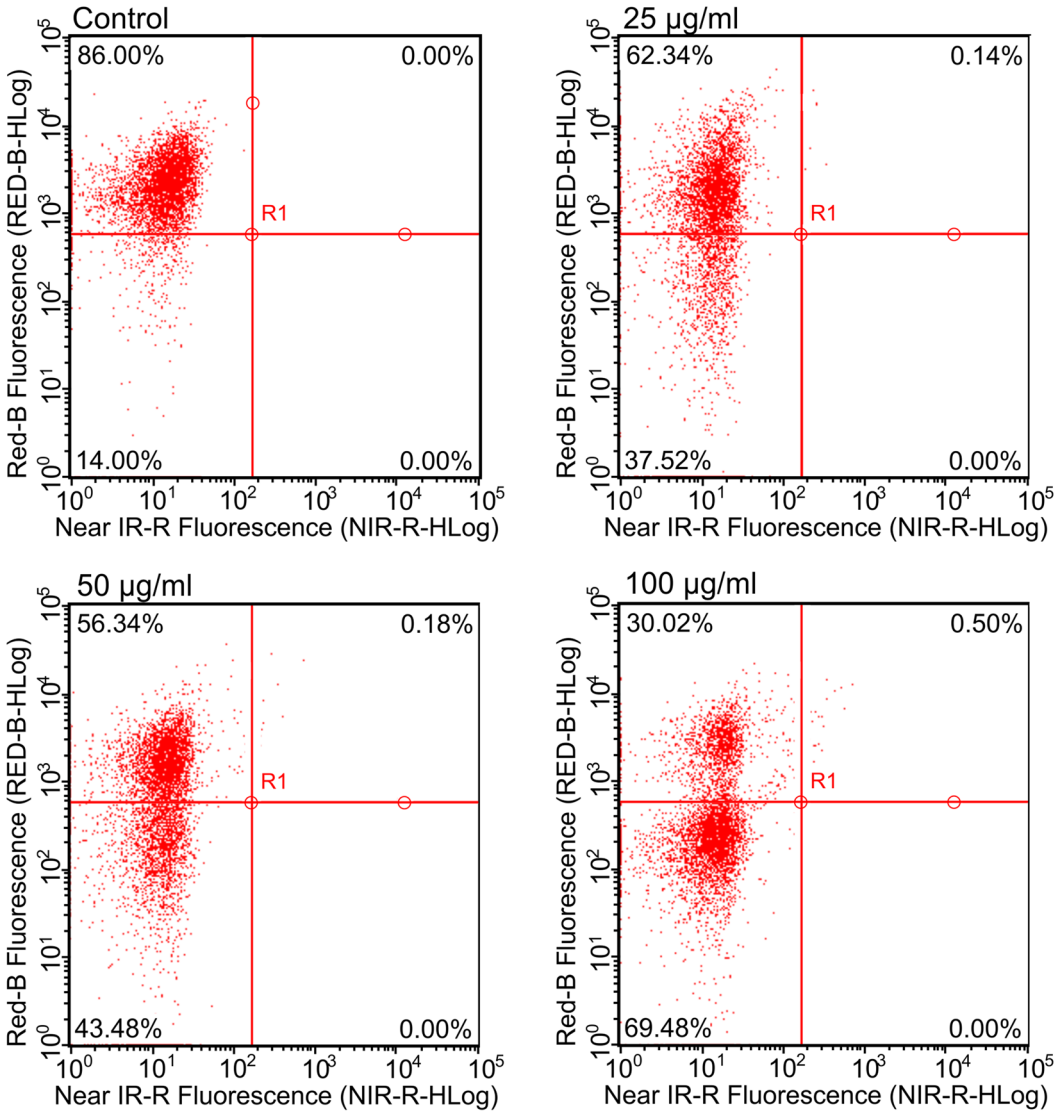
Flow cytometry analysis of viable and dead cells using mixture fluorochromes of Hoechst 33342 and Propidium iodide. The upper left quarter gates viable cells; the lower left quarter gates dead cells.

The flow cytometry analysis of *C. albicans* cells treated with Venetin-1 performed using a mixture of Hoechst 33342 and Propidium iodide showed significant changes in the level of dead cells in cultures treated with Venetin-1 in comparison to the control culture (Fig. 2). The upper left side of the dot-plot shows that 86% of cells were viable in the control culture. After the incubation with Venetin-1, the level decreased to 62.34%, 56.34%, and 30.02% in cultures after treatment with active compound at 25 µg mL^−1^, 50 µg mL^−1^, and 100 µg mL^−1^, respectively.

### SEM analysis of *C. albicans* cells after treatment with Venetin-1

**Figure 3:**
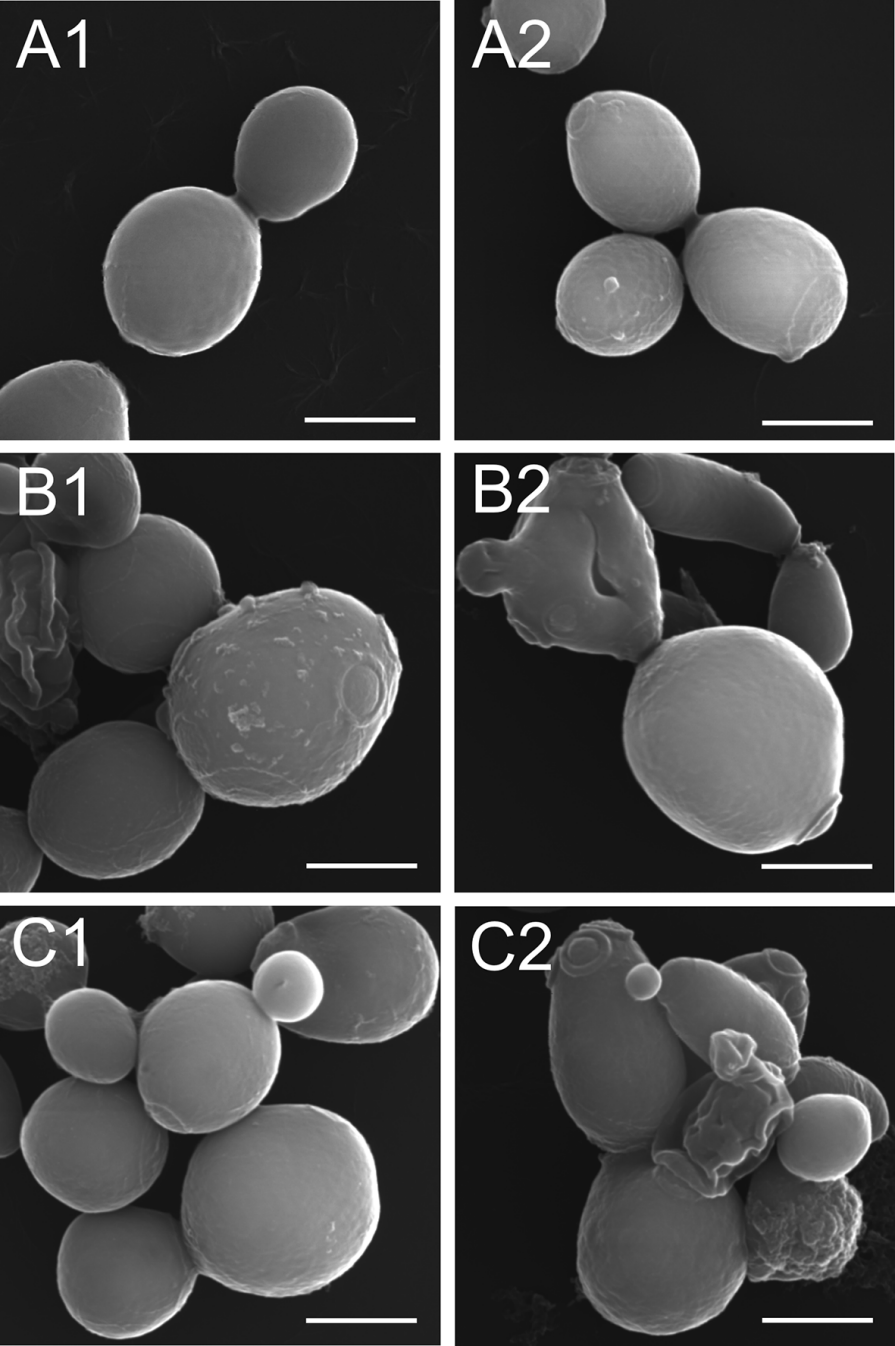
SEM images of *C. albicans* cells. (**A1**,**A2**) *C. albicans* cells of the control culture, (**B1**,**B2**) *C. albicans* cells after incubation with Venetin-1 at 50 µg mL^−1^, (**C1**,**C2**) at 100 µg mL^−1^. Images (**B1**–**C2**) show enlarged and deformed cells. The scale bar represents 2 µm.

Control *C. albicans* cells and those treated with Venetin-1 were imaged by SEM. The morphology analysis showed that the cells of the control culture had an oval shape and a rough but regular cell wall (Fig. 3A1–A2). After the incubation with Venetin-1 at a protein concentration of 50 µg mL^−1^, the yeast cells underwent visible changes. The cells exposed to the active compound clearly enlarged compared to the control cells (Fig. 3B1–B2). In addition, Fig. 3B1 shows cell with exfoliated of the cell wall outer layer, and cell shape deformation is visible in Fig. 3B2. After the treatment with Venetin-1 at 100 µg mL^−1^ (Fig. 3C1–C2), enlarged cells (Fig. 3C1–C2), as well as cells with numerous division scars and shrunken forms were observed (Fig. 3C2). The average cell size was 5.67 µm in the control cell culture, 7.28 µm in the culture treated with Venetin-1 at the concentration of 25 µg mL^−1^, 7.1 µm in the variant with the concentration of 50 µg mL^−1^, and 7.37 µm in the treatment with the concentration of 100 µg mL^−1^ (Supplementary Information Table S1, Fig. S1). Parametrical data distribution was determined with the Shapiro–Wilk test (results in Supplementary Table S1). The Levene test results (F(3, 795) = 12.3181; p < 0.001) indicated non-homogenous variances. Statistical significance was analyzed with the post-hoc Tukey HSD test and one-way ANOVA: F(3, 795) = 219.12; p < 0.001.

### DIC and Cryo-SEM analysis of *C. albicans* autophagy

**Figure 4:**
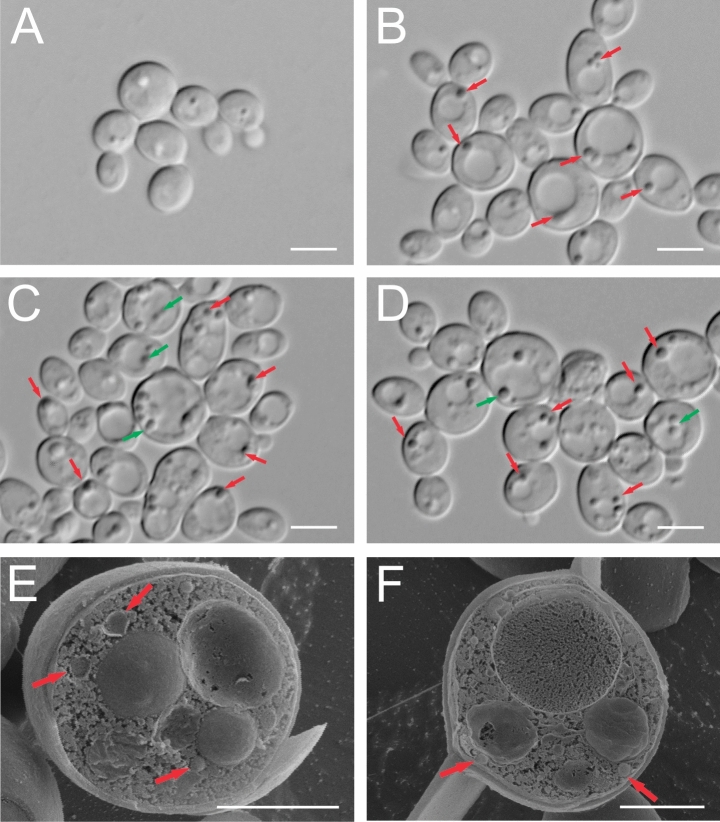
DIC and Cryo-SEM images of *C. albicans* cells: (**A**–**D**) DIC images: (**A**) control culture cells, (**B**) cells after incubation with Venetin-1 at the concentration of 50 µg mL^−1^, (**C**,**D**) at the concentration of 100 µg mL^−1^. (**E**,**F**) Cryo-SEM images of cells after treatment with Venetin-1 at 100 µg mL^−1^. The red arrows indicate autophagosomes; the green arrows mark autophagic bodies. The scale bar corresponds to 2 µm.

The *C. albicans* control culture cells and the Venetin-1-treated cells were imaged by DIC. The control culture cells had an oval shape with a vacuole constituting no more than half of the cell lumen (Fig. 4A). After the exposure to the preparation at a concentration of 50 µg mL^−1^, enlarged cells with clearly enlarged vacuoles occupying most of the cell lumen were observed (Fig. 4B). Small vesicles (autophagosomes) (marked with red arrows in Fig. 4B) were noticeable at the outer edge of the vacuole. After the incubation of the yeast cells with Venetin-1 at a concentration of 100 µg mL^−1^, in addition to enlarged vacuoles and autophagosomes (marked by red arrows), there were vesicles penetrating the vacuole or already present inside (marked with green arrows in Fig. 4C,D; autophagic bodies). After the exposure to the preparation at a higher concentration (100 µg mL^−1^), there were definitely more vesicles around the vacuole than in the treatment with the complex at a concentration of 50 µg mL^−1^. When absorbed by vacuoles, they lost their regular round shape.

Images E and F in Fig. 4 show a cross section of a *C. albicans* cell after the treatment with Venetin-1 at a concentration of 100 µg mL^−^^1^ captured by Cryo-SEM. Images E and F present vacuoles and autophagosomes (marked with arrows). Large vacuoles are visible in Fig. 4E and an enlarged cell nucleus is shown by image Fig. 4F.

### Detection of autophagy using fluorescence microscopy

**Figure 5:**
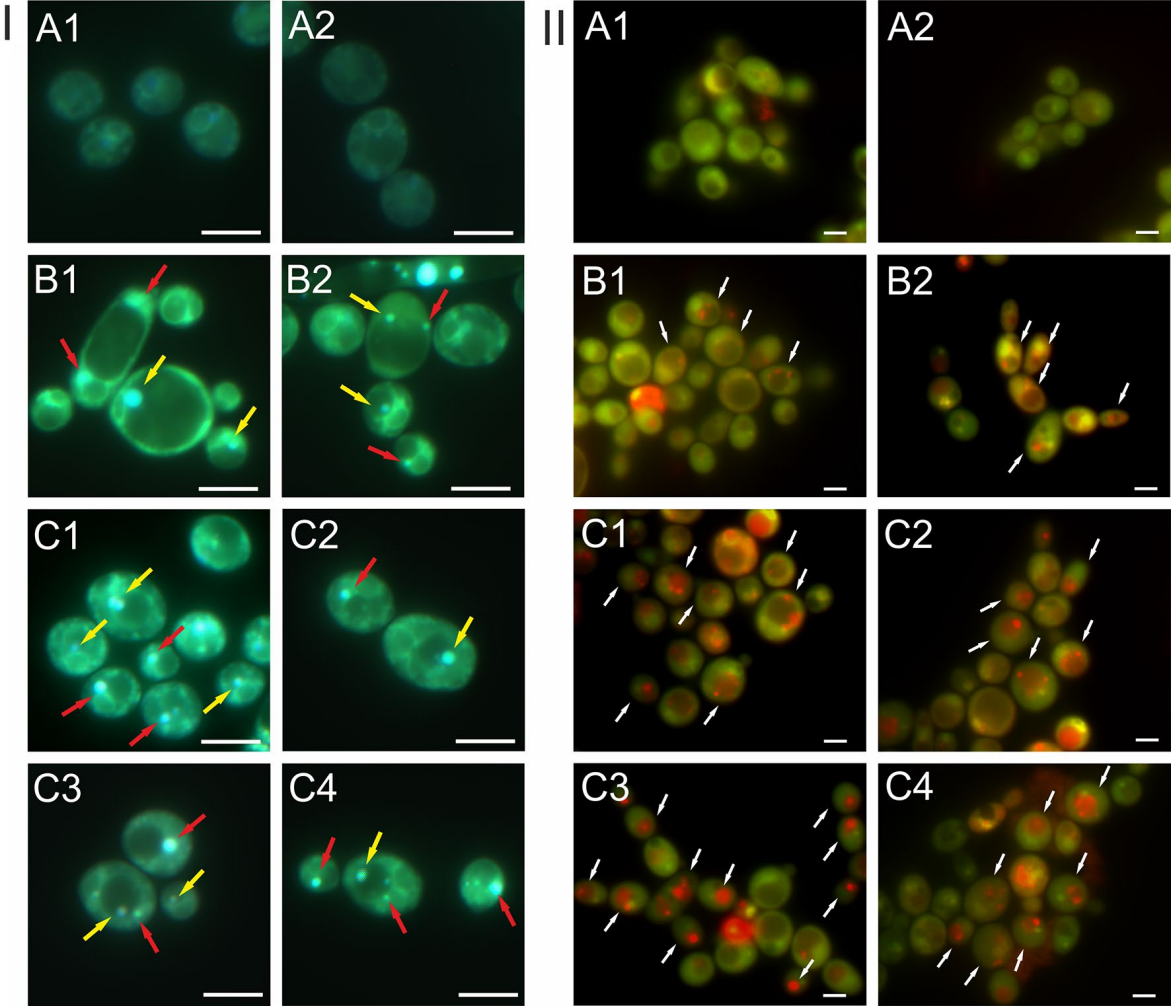
Autophagy in *C. albicans* cells visualized with different fluorochromes. (**I**) Staining with Quinacrine dihydrochloride: A1–A2—*C. albicans* control culture cells; B1–B2—*C. albicans* cells after incubation with Venetin-1 at the concentration of 50 µg mL^−1^; C1–C4—at the concentration of 100 µg mL^−1^. The red arrows indicate autophagosomes, yellow arrows—autophagic bodies. (**II**) Staining with acridine orange: A1–A2—*C. albicans* control culture cells; B1–B2—*C. albicans* cells after incubation with Venetin-1 at the concentration of 50 µg mL^−1^; C1–C4—at the concentration of 100 µg mL^−1^. The white arrows indicate cells with autophagic vesicles with acidic pH. The scale bar corresponds to 1 µm.

The Quinacrine dihydrochloride staining revealed the presence of blue and blue-green vesicles located both inside and outside the vacuoles (Fig. 5I). The staining of cellular structures in the presented images is non-specific. No vesicle fluorescence was observed in the control *C. albicans* culture cells. In the microscopic image, the outline of vacuoles with a normal size only was visible (Fig. 5I A1–A2). The *C. albicans* cells treated with Venetin-1 at a concentration of 50 µg mL^−1^ were characterized by larger cell and vacuole sizes than in the untreated culture. Both autophagosomes, i.e. vesicles located outside the vacuolar lumen (indicated by red arrows), and autophagic bodies, i.e. vesicles located inside the vacuoles (indicated by yellow arrows), were visible (Fig. 5I B1, B2). In turn, the *C. albicans* cells treated with Venetin-1 at a concentration of 100 µg mL^−1^ exhibited a greater amount of both types of autophagic vesicles: autophagosomes (red arrows) and autophagic bodies (yellow arrows). The size of the vacuoles did not differ from the normal size (Fig. 5I C1–C4).

The above observations were confirmed by the analysis of *C. albicans* cells performed using acridine orange (AO), which stains acidic cell compartments orange or red. This fluorochrome is used to stain autophagic cells with characteristic red fluorescent vesicles (Fig. 5II). The microscopic image of the control culture showed green cells with a normal size (Fig. 5II A1–A2). In turn, small red spots were visible in the yeast cells incubated with Venetin-1 at a concentration of 50 µg mL^−1^, indicating the presence of acidic vesicles (pointed by white arrows) (Fig. 5II B1–B2). The *C. albicans* cells treated with Venetin-1 at a concentration of 100 µg mL^−1^ had a significantly greater number of red vesicles (indicated by white arrows). In addition, they were larger and more intensely stained than in cells treated with a lower concentration of Venetin-1 (Fig. 5II C1–C4). The average percentage of cells with visible red autophagic bodies was 9.44% in the control cell culture and was higher in samples treated with Venetin-1: 16.25% in the culture treated with the concentration of 25 µg mL^−1^, 33.85% in the treatment with 50 µg mL^−1^, and 62.70% in the variant with 100 µg mL^−1^ (Supplementary Information Table S2, Fig. S2). The parametrical data distribution was confirmed with the Shapiro–Wilk test (results in Supplementary Table S2). Homogenous variances were indicated by the Levene test: F (3, 8) = 1.1395; p = 0.313115. Statistical significance was analyzed with the post-hoc Tukey HSD test and one-way ANOVA: F(3, 8) = 423.51; p = 0.000.

### TEM analysis of *C. albicans* cell autophagy

**Figure 6:**
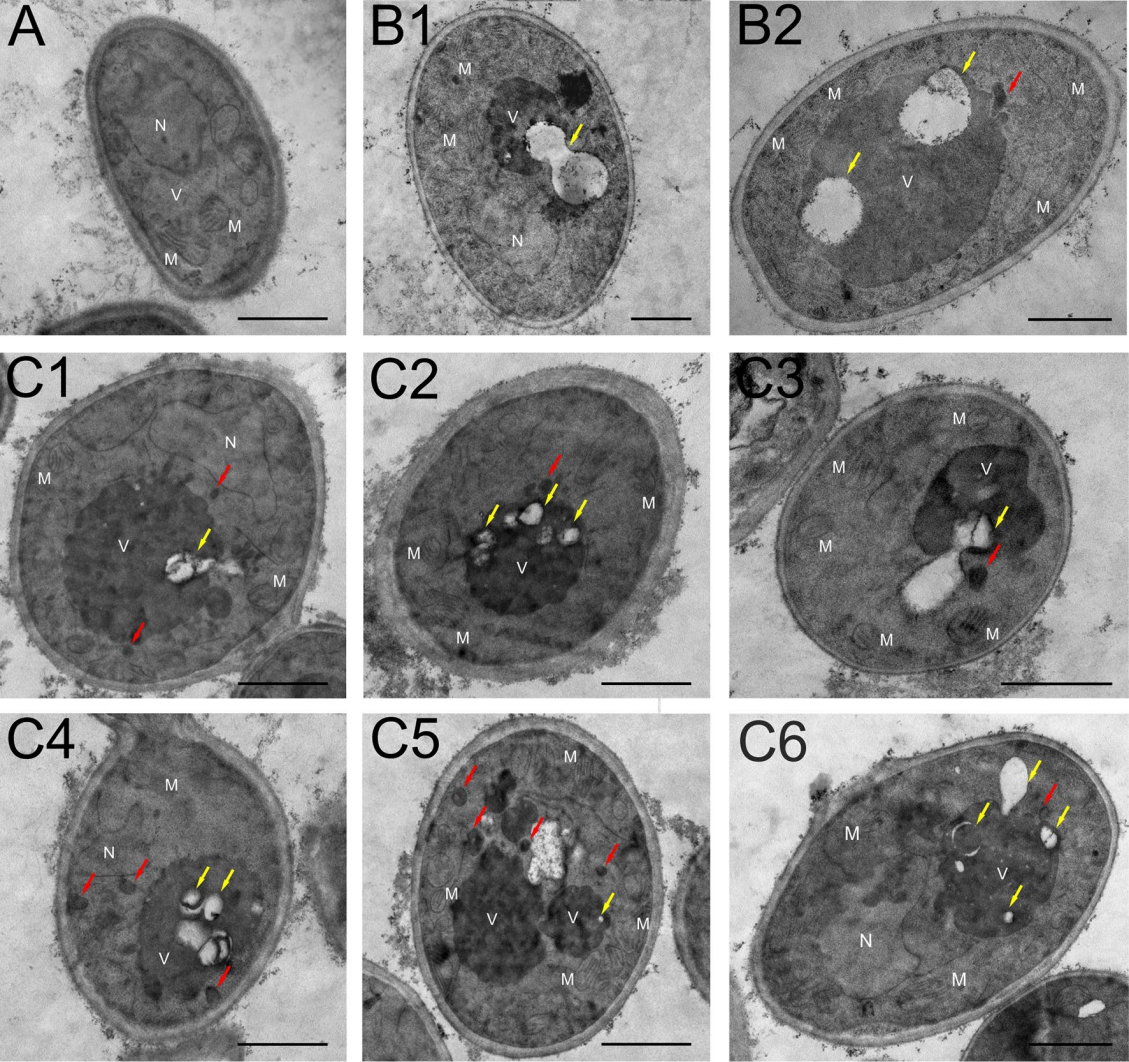
TEM imaging of *C. albicans* cells: (**A**) control cell; (**B1**,**B2**) cells after incubation with Venetin-1 at the concentration of 50 µg mL^−1^; (**C1**,**C6**) at the concentration of 100 µg mL^−1^. *V* vacuole, *N* nucleus, *M* mitochondrion, red arrows—autophagosomes; yellow arrows—autophagic bodies. The scale bar corresponds to 1 µm.

The ultrastructure of *C. albicans* cells after the application of Venetin-1 was analyzed with the use of transmission electron microscopy. The control cells were characterized by a regular cell wall and normal intracellular structure with visible organelles, i.e. the nucleus (N), a single vacuole (V), and mitochondria (M) with no signs of autophagy (Fig. 6A). In turn, numerous autophagic vesicles of two types were visible in the *C. albicans* cells incubated with Venetin-1 at the concentrations of 50 µg mL^−1^ and 100 µg mL^−1^. Moreover, the complex-treated cells had larger vacuoles and more mitochondria than the control culture cells.

The TEM technique allowed observation of several steps of the autophagy process. Mature and completely closed autophagosomes (indicated by red arrows) transporting their contents to the vacuole were visible in the cell cytoplasm (Fig. 6B1–B2,C1–C6). Subsequently, the stages of autophagosome fusion with vacuoles, release of the internal vesicle into the vacuole lumen, and formation of autophagic bodies were captured (Fig. 6B1,C3,C6; autophagic bodies are pointed by yellow arrows).

The vesicles visible in the cytoplasm varied in size, which may indicate the occurrence of different types of macroautophagy. The larger autophagosomes are typical of non-selective macroautophagy, where the vesicle contains bulk cytoplasm, and the smaller ones are typical of the selective process, where specific structures of the yeast cell constitute the contents. However, in the *C. albicans* cells treated with Venetin-1 at the concentration of 50 µg mL^−1^, all autophagy vesicles were noticeably larger than in the cells incubated with Venetin-1 at the concentration of 100 µg mL^−1^. The presented pictures are representative of the 30 images obtained.

### Visualization of mitochondria using fluorescence microscopy

**Figure 7:**
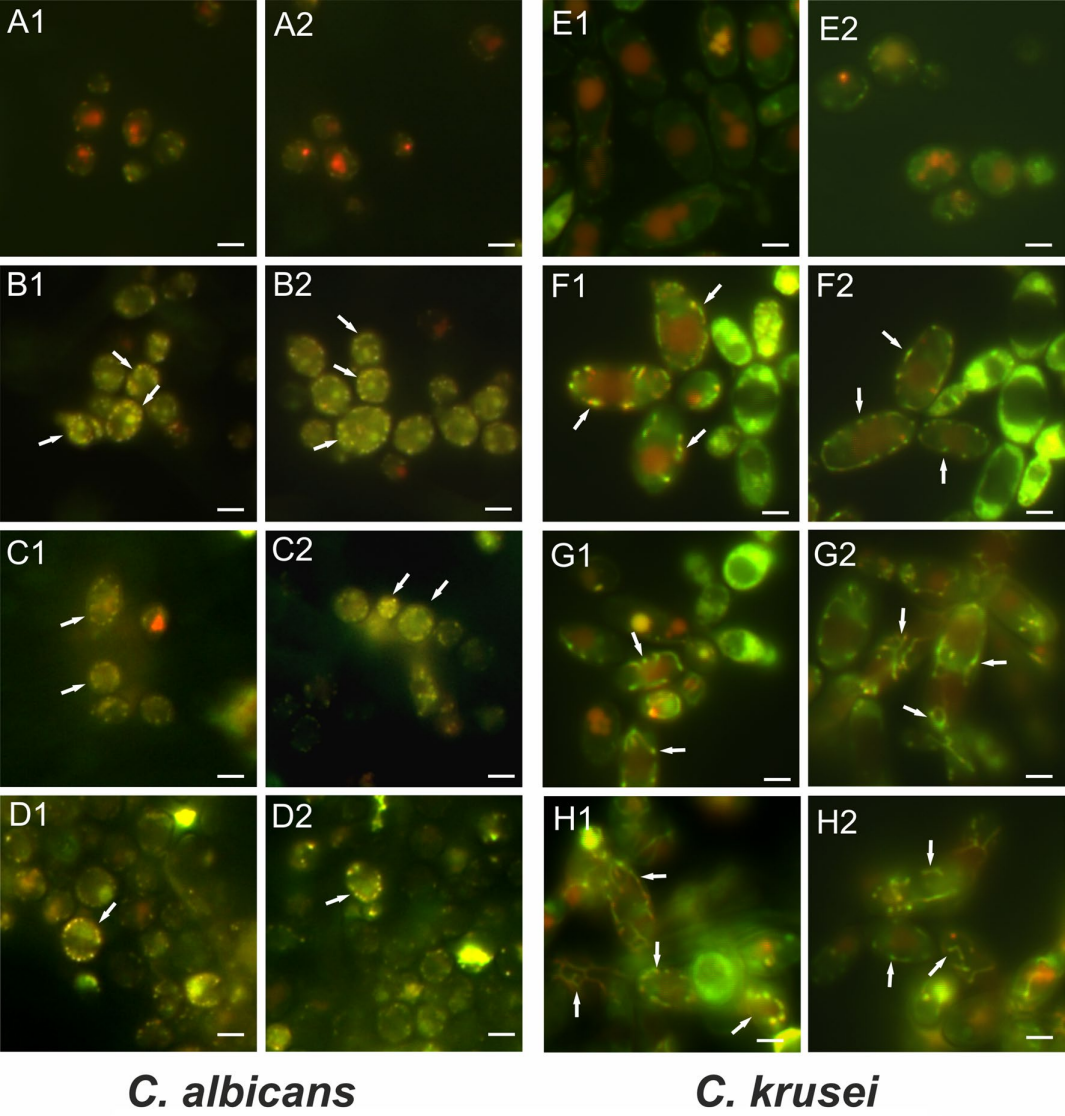
Visualization of mitochondria in *C. albicans* and *C. krusei* cells after staining with FUN-1: (**A1**,**A2**,**E1**,**E2**) control culture cells; (**B1**,**B2**,**F1**,**F2**) cells after incubation with Venetin-1 at the concentration of 25 µg mL^−1^; (**C1**,**C2**,**G1**,**G2**) at the concentration of 50 µg mL^−1^; (**D1**,**D2**,**H1**,**H2**) at the concentration of 100 µg mL^−1^. The arrows indicate cells with stained mitochondria. The scale bar corresponds to 3 µm.

The Live/Dead Yeast Viability Kit is used as a standard to determine the metabolic activity of fungal cells. The analysis is based on the ability of cells to metabolize the FUN-1 dye, as a result of which red structures are visible in cell vacuoles. It has been used in our previous studies^49^. The use of this kit facilitated additional visualization of mitochondria (Fig. 7). The labeling of these organelles was a non-specific effect. The *C. albicans* control culture was characterized by metabolically active cells with red structures inside the vacuole. In turn, no structures other than vacuoles were visible in the control cells (Fig. 7A1–A2). After the incubation of the *C. albicans* cells with Venetin-1 at the protein concentrations of 25, 50, 100 µg mL^−1^, spot yellow fluorescence of numerous mitochondria was observed. These organelles were arranged in a characteristic way forming a ring under the surface of the membrane (Fig. 7B1–B2,C1–C2,D1–D2; pointed by arrows).

To confirm our observations of mitochondria, another strain of *Candida*, *C. krusei*, was incubated with Venetin-1 as well. The effect of mitochondrial fluorescence after the treatment with Venetin-1 in this case was stronger. The control cells showed no stained mitochondria (Fig. 7E1–E2). In turn, in the cells incubated with the active compound, yellow-green fluorescing mitochondria were clearly visible. After the treatment of *C. krusei* cells with Venetin-1 at a concentration of 25 µg mL^−1^, their mitochondria were small and visible as spots arranged on the periphery of the cells (Fig. 7F1–F2; pointed by arrows). In the case of cells treated with Venetin-1 at the concentrations of 50 and 100 µg mL^−1^, elongated forms of brightly fluorescent mitochondria were observed (Fig. 7G1–G2,H1–H2; pointed by arrows).

### Cryo-TEM analysis of Venetin-1

**Figure 8:**
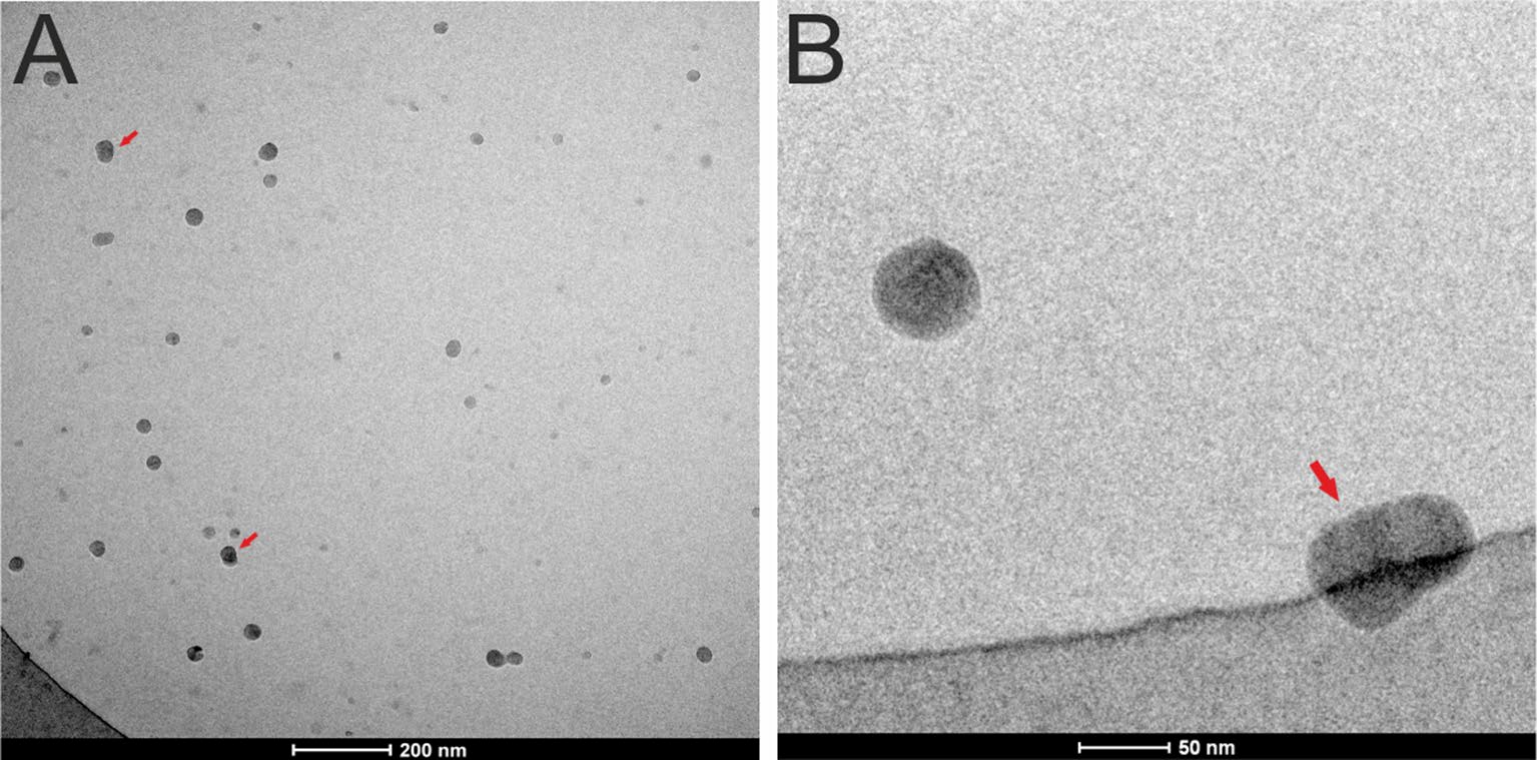
Cryo-TEM visualization of Venetin-1. The nanoparticles are visible as circular dark structures sometimes merging into a double form (indicated by red arrows).

The analysis of the Venetin-1 complex by Cryo-TEM confirmed the nanoparticle size determined by the DLS analysis^25^. The nanoparticles were visible under the microscope as spherical forms, which sometimes coalesced to form double forms indicated by red arrows in Fig. 8A,B.

### Dynamic light scattering domain-specific characterization of Venetin-1

**Figure 9:**
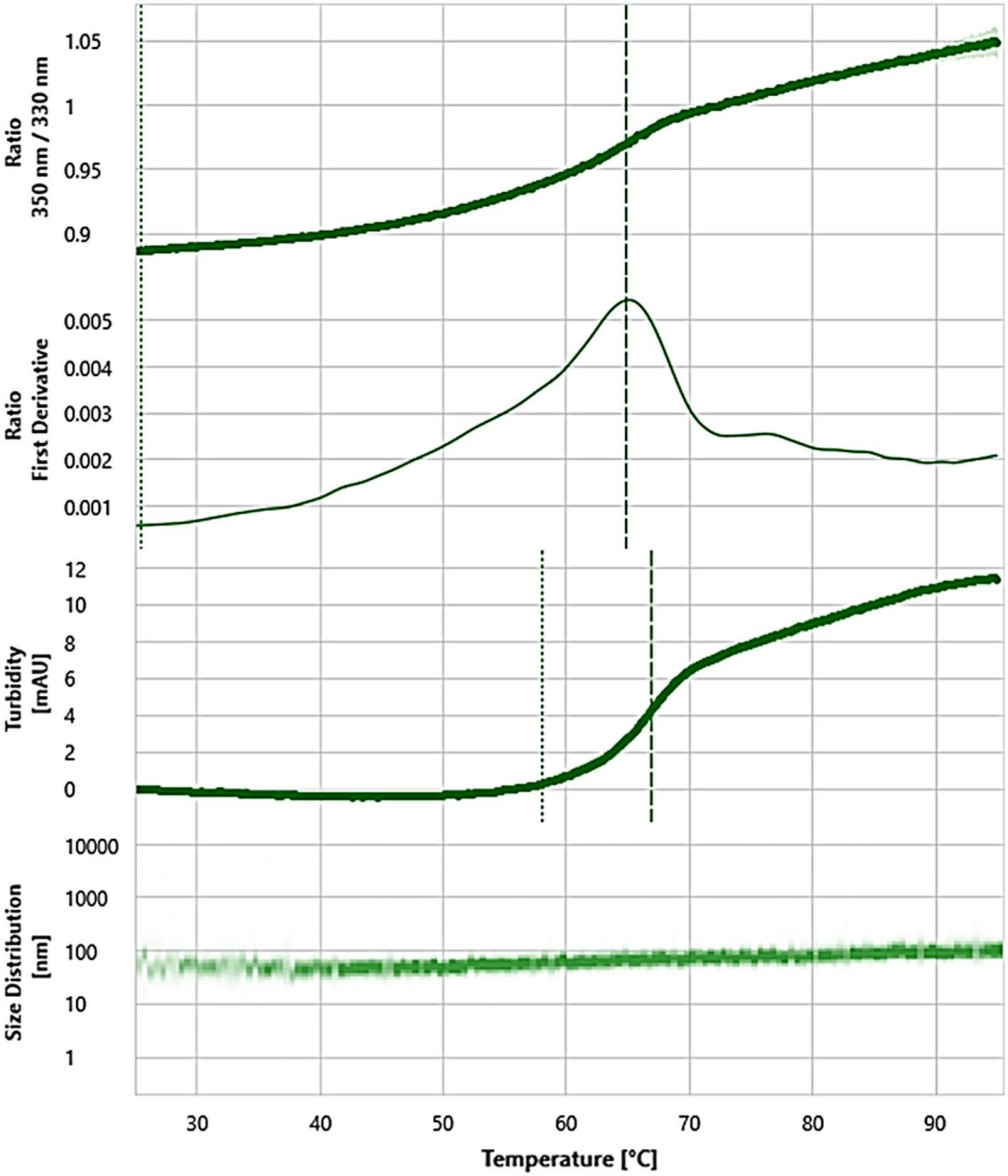
Prometheus Panta melting profile of Venetin-1 nanoparticles. From above: Panel 1—ratio 350/330 nm corresponding to the conformational changes in the nanoparticles. Panel 2—First derivative of the ratio 350/330 nm; Panel 3—turbidity displaying the aggregation; Panel 4—size distribution analysis.

Following the analysis of Venetin-1 size (58.23 ± 3.93 nm)^25^ conducted with the use of Prometheus Panta, a temperature melting experiment with a temperature gradient from 25 to 95 °C and a heating ramp of 1 °C/min was performed. As shown in Fig. 9, the nanoparticles display single 350/330 nm transition at a temperature of 64.95 °C ± 0.08 °C. This transition very likely corresponded to the melting point of the analyzed nanoparticles. Moreover, it was determined that, following the conformational change represented by the fluorescence ratio 350/330 nm, the analyzed nanoparticles underwent aggregation with the Tagg 66.92 ± 0.16 °C and aggregation onset 58.97 ± 1.15 °C. Interestingly, the DLS analysis on the fly clearly showed an increase in the overall nanoparticle size resulting in formation of larger 100 nm nanoparticles at a temperature above the melting point (Fig. 9, Table 1).

**Table 1:** Prometheus Panta melting profile parameters.

Annotations	Ratio ↑				Turbidity ↑					
	IP #1 (°C)		ON (°C)		IP #1 (°C)		ON (°C)		Turbidity average	
Sample ID	ø	σ	ø	σ	ø	σ	ø	σ	ø	σ
Venetin-1	64.95	0.08	25.50	0.66	66.92	0.16	58.19	1.57	9.0	0.3

### Determination of Venetin-1 zeta potential

**Figure 10:**
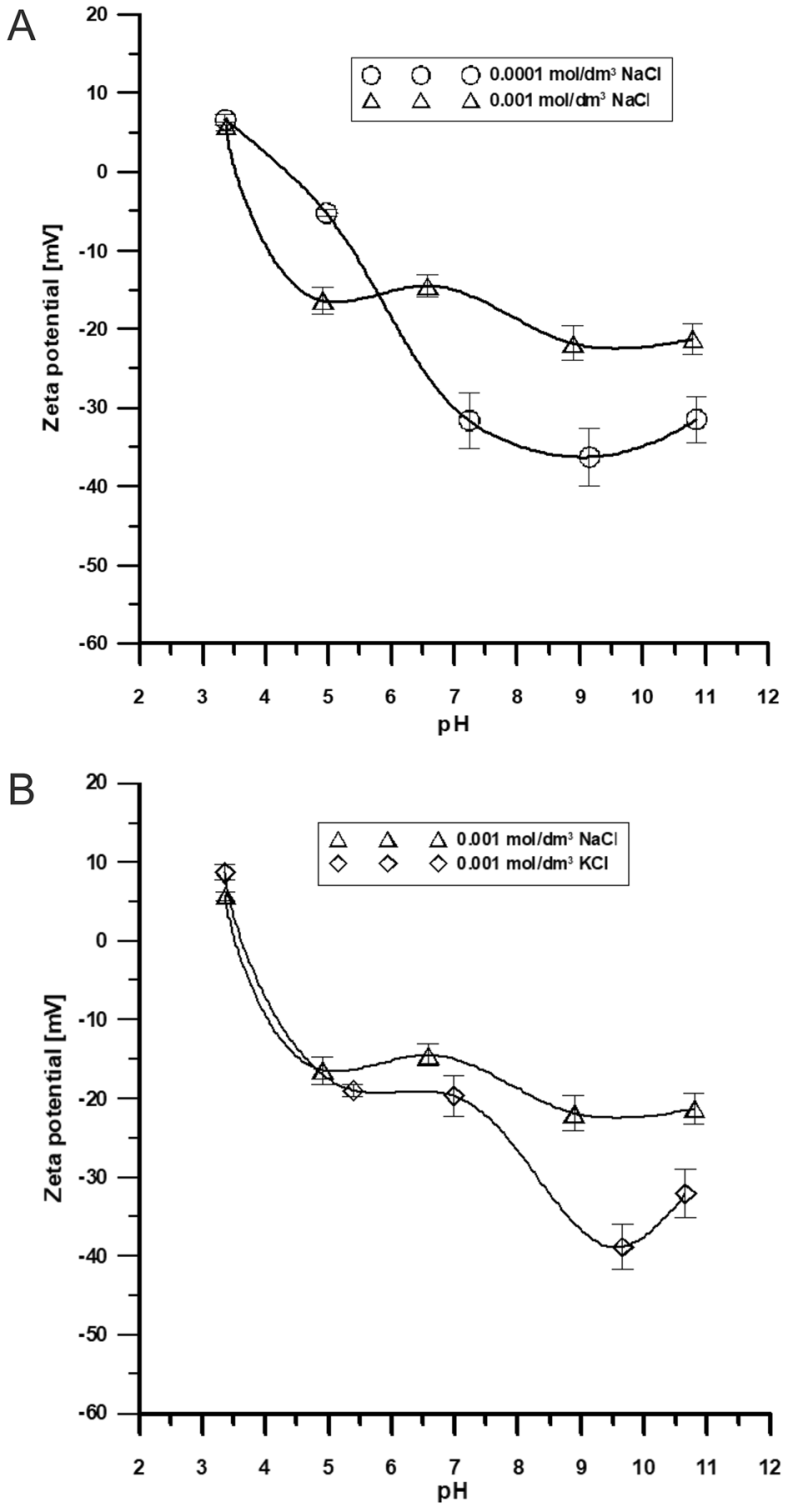
(**A**) Dependence of ζ potential of Venetin-1 in the pH function in 0.001 M and 0.0001 M NaCl solutions. (**B**) Dependence of ζ potential of Venetin-1 in the pH function in 0.001 M NaCl and 0.001 M KCl solutions.

Five measurements of electrophoretic mobility, conductivity, and zeta potential were made for each solution (Table 2) (Fig. 10A,B). The zeta potential of the tested samples, in the tested pH ranges from 3 to 12 and different electrolyte concentrations, ranged from 10 to − 40 mV. In the majority of the ranges tested, the selected systems are unstable in the colloidal form and may be delaminated. Stable systems at the absolute potential lower than − 30 mV were obtained only for the following samples: V-1/0.0001 M NaCl at pH = 7, 9, 11; V-1/0.001 M KCl at pH < 8 (Fig. 10B). The comparison of the results for 0.001 mol/dm^3^ NaCl and 0.001 M KCl showed very different absolute values for pH > 4 at the same electrolyte concentrations (Fig. 10A,B). Therefore, the surface potential of Venetin-1 was found to exert the main impact on the zeta potential.

**Table 2.** Electrophoretic mobility, conductivity, and pH of Venetin-1 in 0.001 M NaCl, 0.0001 M NaCl, and 0.001 M KCl.

pH	Electrophoretic mobility [ms/Vs]	Conductivity[mS/cm]
0.001 M NaCl		
3.37	0.412	0.473
4.91	− 1.236	0.239
6.58	− 1.480	0.209
8.89	− 1.505	0.226
10.8	− 1.843	0.228
0.0001 M NaCl		
3.36	0.490	0.329
4.97	− 0.441	0.109
7.24	− 2.094	0.135
9.15	− 2.370	0.127
10.85	− 2.978	0.098
0.001 M KCl		
3.36	0.729	0.401
5.39	− 1.175	0.252
6.99	− 1.262	0.299
9.46	− 2.180	0.238
10.66	− 2.482	0.357

As shown in Fig. 10A,B, the studied system had the highest absolute values of the zeta potential at the lowest electrolyte concentration and the lowest absolute values at the highest electrolyte concentration. It was found that the zeta potential decreased with the increasing electrolyte concentration, resulting from the dissociation of surfaces subjected to ionization on Venetin-1. In the examined systems, the zeta potential decreased with increasing pH. The state of the solid surface in which the amounts of positive and negative charges in the diffuse layer of the electric double layer (edl) are equal to each other is called the isoelectric point of the solid surface (IEP—IsoElectric Point) pH_IEP_. Then, the resultant charge of the diffuse layer of the double electrical layer is zero. Since the concentration of potential-forming ions (H^+^ and OH^−^) depends on the pH of the solution, the pH_IEP_ point corresponds to a precisely determined pH value. The pH_IEP_ values of Venetin-1 are below pH 6 for NaCl (Fig. 10A) and pH_IEP_ = 4 for KCl (Fig. 10B). This means that in a KCl solution above pH = 4, the zeta potential becomes positive and negative below, which may affect the electrostatic interactions with other particles surrounding Venetin-1.

### Inhibitory potency against selected serine proteases

**Figure 11:**
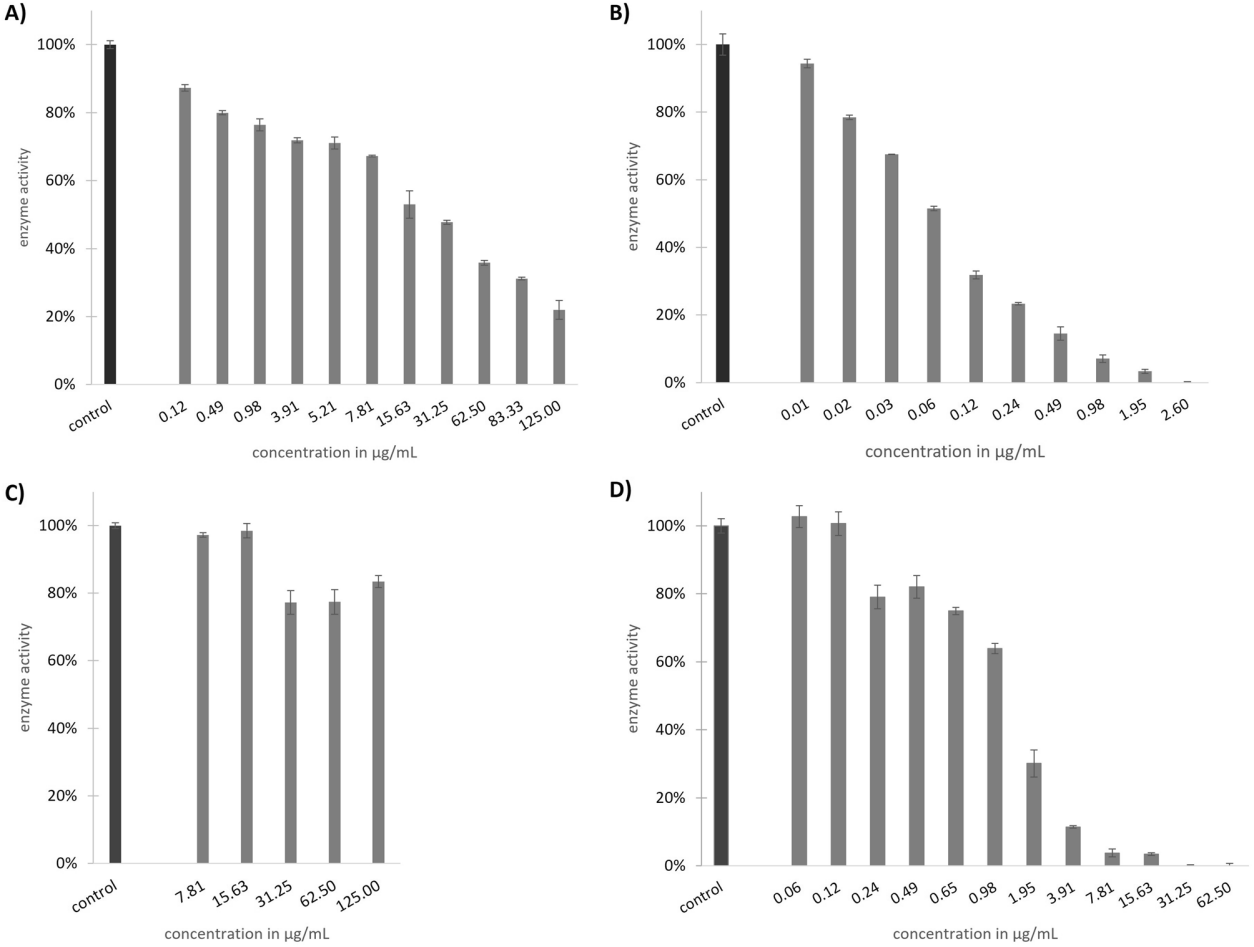
Proteolytic activity of matriptase-1 (**A**) and matriptase-2 (**B**), trypsin (**C**), and chymotrypsin (**D**) after treatment with Venetin-1 *versus* the control sample without the inhibitor.

The inhibitory activity of Venetin-1 was examined against selected serine proteases, i.e. two digestive enzymes: trypsin and chymotrypsin, and three transmembrane proteases: matriptase-1 (MT1), matriptase-2 (MT2), and furin. In this study, Venetin-1 exhibited moderate inhibitory potency towards MT1 (IC_50_ value of 176.608 ± 91.164 μg mL^−1^; Fig. 11A), while the inhibition of MT2 was much more remarkable with IC_50_ 0.06 ± 0.01 μg mL^−1^ (Fig. 11B). The inhibition of furin was marginal at the highest concentration of 250 μg mL^−1^,and this enzyme retained over 70% of its initial proteolytic activity. Due to the close structural homology but distinct physiological functions, selective inhibitors of matriptases are of great interest nowadays. However, further more detailed research is essential to clearly determine the selectivity of Venetin-1 towards MT1 and MT2, which is beyond the scope of this paper. Additionally, it was shown that the tested compound inhibited chymotrypsin (IC_50_ 1.26 ± 0.14 µg mL^−1^) and was inactive towards trypsin (Fig. 11C,D).

## Discussion

Annelids inhabit soil rich in microorganisms and have developed a unique ability to survive in this environment; therefore they represent a group of animals that is intensively studied by biologists in order to find new molecules with potential therapeutic applications. These model invertebrates are crucial for elucidation of the mechanisms of important biological and developmental processes in organisms. Earthworms as a model organism are inexpensive and, importantly, ethically uncontroversial^63^. Currently, bearing in mind the lack of sufficient effectiveness and selective action of synthetic preparations, the pharmaceutical industry is returning to natural bioactive substances that may prove effective where modern medicine fails^64^.

Venetin-1, previously characterized as a protein-polysaccharide fraction, also showed inhibitory activity against *C. albicans* ATCC 10231 and *C. krusei* ATCC 6258. Venetin-1 disrupted cell division and led to fungal cell death^49^. The preparation induced changes in the fungal cell wall structure and in the nanomechanical properties of the cell wall, as evidenced by atomic force microscopy (AFM)^50^. Upon incubation of *C. albicans* with Venetin-1, mitochondrial DNA migrated towards nuclear DNA. Both genetic materials combined into one nuclear structure, which was the beginning of the apoptosis process. It was observed that the analyzed compound affected the expression of oxidative stress proteins^51^. The protein part of the nanoparticle was shown to consist mainly of two lysenin proteins^50^. The presence of carbohydrate compounds in the preparation was confirmed as well. The analyzed compound did not show endotoxicity and cytotoxicity in relation to normal human skin fibroblasts^49^.

In previous analyses, Venetin-1 was shown to cause *C. albicans* cell death via necrosis and apoptosis^49,51^. Generally, there are three main types of cell death: apoptosis, necrosis, and autophagy. Apoptosis, or programmed cell death, is an active process that requires activation of many genes and energy expenditure. Necrosis, in contrast to apoptosis, is a passive and pathological process. This type of death occurs under the influence of physical, chemical, and biological factors after exceeding the threshold value of cell immunity. Autophagy is a mechanism referred to as type II programmed cell death^65^. In this process, proteins with a long half-life and other components of organelles are directed to vacuoles, where they are degraded^66,67^. In the present study, autophagy was observed during the analysis of the action of Venetin-1.

Autophagy, which is a process specific for eucaryotic cells, has three main forms: chaperone-mediated autophagy, microautophagy, and macroautophagy. Macroautophagy is the most common type and is activated to degrade damaged organelles and proteins. A small amount of cytoplasm containing damaged structures is enveloped by a double-layered vesicle creating an autophagosome and transported to the vacuole. The outer layer of the autophagosome fuses with the vacuolar membrane, and the inner layer with its contents passes inside the vacuole as an autophagosomal body, where it is degraded by vacuolar enzymes^68–70^.

The presented fluorescent microscopy and transmission electron microscopy analyses of vacuoles convincingly showed the process of autophagy, i.e. engulfment of vesicles after the incubation with Venetin-1. The *C. albicans* cells treated with Venetin-1 had significantly bigger vacuoles than the control cells. Those changes may have a source in the autophagy process, where every autophagosome adds its membrane to the vacuolar membrane. This additional membrane intake is not balanced by outflow and leads to vacuole enlargement and dilution of intramembrane contents^71–74^.

The process of autophagy usually involves leading the cell through unfavorable living conditions. Autophagy may be a strategy for cell survival in stress conditions such as starvation, hypoxia, or the use of chemotherapeutics. The mechanism of autophagy is activated to obtain an additional source of energy.

Autophagy is an essential process for cell survival, as it removes damaged or abnormal organelles and molecules that can disrupt cell homeostasis. After the action of Venetin-1, oxidative stress was observed, which led to damage to intracellular structures. There are studies that prove that ROS (reactive oxygen species) play a regulatory role in the process of autophagy—the appearance of ROS activates this process. Activation of the autophagy process by ROS aims to minimize ROS-induced damage^75^.

Mitochondrial ROS can be beneficial or harmful to cells depending on their concentration and location^76^. Mitophagy, where inefficient mitochondria are degraded, is a type of selective autophagy. Mitochondria provide most of the energy required by the cell. Energy production by mitochondria is accompanied by generation of ROS. An increased amount of mitochondrial ROS damages cell components, including the mitochondria. This leads to various pathological changes and results in cell death^76^. An important process for the cell is the elimination of overactive or damaged mitochondria in order to maintain mitochondrial homeostasis. This removal must take place in a timely manner so as not to cause further cell damage^77,78^.

After the treatment of the *C. albicans* cells with Venetin-1, these overactive mitochondria were enlarged and showed stronger fluorescence in the microscopic image. In addition, the *C. krusei* strain was analyzed in this respect. It turned out that the effect was repeated and was additionally stronger than in the case of *C. albicans*, and the mitochondria were visible as large, elongated structures located at the edge of the cell. Enlarged mitochondria and increased production of ROS were observed in our previous studies^51,52^. There are data reporting that mitochondrial outer membrane proteins have been identified as mitophagy receptors in yeast^77^. The patch of vacuoles and mitochondria is an interesting and increasingly often discussed topic in the characterization of yeast biology. In *C. albicans* cells, the Mcp1 vacuole and mitochondrial patch protein (vCLAMP) is involved in maintaining mitochondrial function and mitophagy^77^. The observed morphological and proteomic changes in the *C. albicans* cells after the treatment with Venetin-1^51^ suggest occurrence the mitophagy, which will be analyzed in further research.

SEM micrographs of *C. albicans* cells after treatment with the test preparation were presented in an earlier publication^51^. In those studies, we showed cell deformation and changes in the surface of the cell wall, which was irregular after the action of the analyzed compound. In the current studies, we have shown significant cell enlargement after the incubation with Venetin-1 in comparison to the control culture cells, which is related to autophagy.

After the action of Venetin-1, numerous post-division scars on the surface of cells were observed. They were similar to those observed in SEM micrographs after exposure to fluconazole, a known drug that disrupts the structure of the cell membrane of *C. albicans*^79^. In our opinion, the cell dividing many times in a short time does not keep up with the production of building compounds required to synthesize the structures of the cell wall in place of scars. In addition, there are disturbances inside the cell, the inhibition of which is associated with the expenditure of cell energy in order to keep the cell alive.

Continuing the multidirectional characterization of the Venetin-1 nanoparticle, we decided to analyze the zeta potential for this complex. An important quantum of particles dispersed in the liquid is its stability, i.e. the ability of the particles to remain in the form of a colloidal dispersion. Dispersions that are unstable may undergo coagulation or sedimentation processes, which result in the delamination of the sample. The zeta potential is the potential occurring in the double layer at the surface of dispersed particles. It is a potential between the dispersant and the fluid layer attached to the surface of the particle. This parameter is used to determine the stability of colloidal systems. It is assumed that the dispersion is stable for an absolute value of zeta potentials >|± 30| V. These conditions were met by some of the analyzed samples. The zeta potential of Venetin-1 depends on the surface potential. The zeta potential was measured in the pH range from 3 to 7 in the 0.001 M NaCl electrolyte and in the pH range from 3 to 8 in 0.001 M KCl. The tested systems were unstable and aggregated, which was confirmed by the particle size measurements made using the Dynamic Light Scattering method.

The present study showed that Venetin-1 additionally exhibits inhibitory activity towards certain proteolytic enzymes, namely matriptase-1 (MT1), matriptase-2 (MT2), and chymotrypsin. Both matriptases are type II transmembrane serine proteases, have trypsin-like specificity, and share high structural similarity but differ in biological activity^80^. MT1 affects the formation and integrity of epithelial tissues and is implicated in various epithelial-derived cancers, including breast, prostate, and ovary tumors^81^. Noteworthy, its increased activity is often regarded as a predictive factor of poor cancer prognosis^82^. MT2 acts as the proteolytic regulator in human iron homeostasis. Mutations in its gene, TMPRSS6, correlate with iron-refractory iron deficiency anemia, while its up-regulated activity may lead to iron overload disorders^83^. The inhibitory activity of Venetin-1 against furin, i.e. a type I transmembrane serine protease involved in the cleavage of many inactive protein precursors in the constitutive secretory pathway, was insignificant^84^.

In addition, the analyses showed that Venetin-1 inhibited strongly chymotrypsin and was inactive towards trypsin. Both proteolytic enzymes are pancreatic serine proteases involved in food digestion displaying different substrate preferences. Trypsin specifically hydrolyzes peptide bonds after basic amino acid residues, such as Lys and Arg, while chymotrypsin cleaves peptide bonds formed by the carboxyl groups of either aromatic Tyr, Phe, and Trp or aliphatic Leu residues.

Inhibition of proteolytic activity is a very important molecular mechanism by which organisms prevent self-injury^85^ and protect themselves against pathogens^86–89^ or predators^90–92^. Of all types of protease inhibitors, serine protease inhibitors (SPIs) are the most common^93^. They are widespread in invertebrates, e.g. in kuruma shrimp (*Marsupenaeus japonicas*), black tiger shrimp (*Penaeus monodon*)^92^, sea anemones^94,95^, scorpions^93,95,96^, hookworms^97^, and numerous poisonous animals—spiders and snails^93,98,99^. SPIs with antifungal activity have been described in the silkworm (*Bombyx mori*)^100^. Studies reported by Lee^92,101^ also showed SPIs produced by the oyster *Crassostrea gigas* having activity against the HIV-1 virus. On the other hand, it has been reported that HIV protease inhibitors have been shown to reduce *C. albicans* cell adherence by inhibiting yeast-secreted aspartic proteases^102,103^. The incidence of candidiasis in HIV-infected patients has been proven to be significantly reduced^104^.

The pharmaceutical industry offers several protease inhibitors, which are used in the treatment of human diseases, such as dabigatran used in the treatment of pulmonary embolism and angiotensin converting enzyme inhibitors (ACEI) used in the treatment of hypertension^92,105,106^. The American Association for the Study of Liver Diseases (AASLD) recommends therapy for genotype-1 chronic hepatitis C virus (HCV) based on serine protease inhibitors together with pegylated interferon α and ribavirin^107^. The proteasome inhibitor bortezomib having also antifungal activity^108,109^ has been approved for clinical treatment of multiple myeloma^110^. It is similar to the Venetin-1 nanoparticle, which inhibits the 20S proteasome and shows antifungal activity at the same time.

Discovering protease inhibitors from the natural environment may have advantages over development of synthetic compounds with such properties There are known natural inhibitors whose mechanism of action is similar to that of Venetin-1, i.e. they are targeted at such intracellular organelles as mitochondria and nucleus and cause oxidative stress and cell death via apoptosis^111–113^. Natural protease inhibitors more easily overcome resistance mechanisms and are more stable and less toxic. These arguments support the advisability of searching for these compounds in nature^92^.

The antifungal effect of earthworm CF has been described mainly in relation to fungi attacking plants^8,22,114^. When CF is used against plant pathogens, the cytotoxicity of the fluid is irrelevant and it can be used in its raw form. However, to be used against fungal infections in humans, the CF preparation must be properly prepared so that it does not show cytotoxicity or endotoxicity. Because our research aims to use Venetin-1 as a chemotherapeutic drug, it had to undergo many laboratory processes to meet the required conditions. Taking into account the properties and multidirectional antifungal and anticancer effects of Venetin-1 and its complex structure, the structural connection of polysaccharides and lysenins seems to be an interesting research topic to be explored using techniques from various scientific disciplines.

In conclusion, the conducted analyses showed that the decrease in the survival of *C. albicans* cells corresponded to changes in the morphology of the cells and cellular organelles, such as vacuoles and mitochondria. Vacuole changes typical of autophagy and enlargement of mitochondria were observed. The zeta potential of the tested preparation was characterized. The aggregation ability and properties of the protease inhibitor type of the Venetin-1 nanoparticle have been proved. The compound, exhibiting inhibitory activities against certain serine proteases and the 20S proteasome, seems to be a promising chemotherapeutic agent to combat *C. albicans* infections in humans and animals.

### Supplementary Information

Supplementary Figure S1.

Supplementary Figure S2.

Supplementary Information.

Supplementary Tables.

## Data Availability

The datasets used and/or analysed during the current study available from the corresponding author on reasonable request.
