# Supplementary figures and images for: Autophagy of Candida albicans cells after the action of earthworm Venetin-1 nanoparticle with protease inhibitor activity
Source: Sci Rep. 2023 Aug 30;13:14228. doi: 10.1038/s41598-023-41281-4 (PMC10468520; doi:10.1038/s41598-023-41281-4)

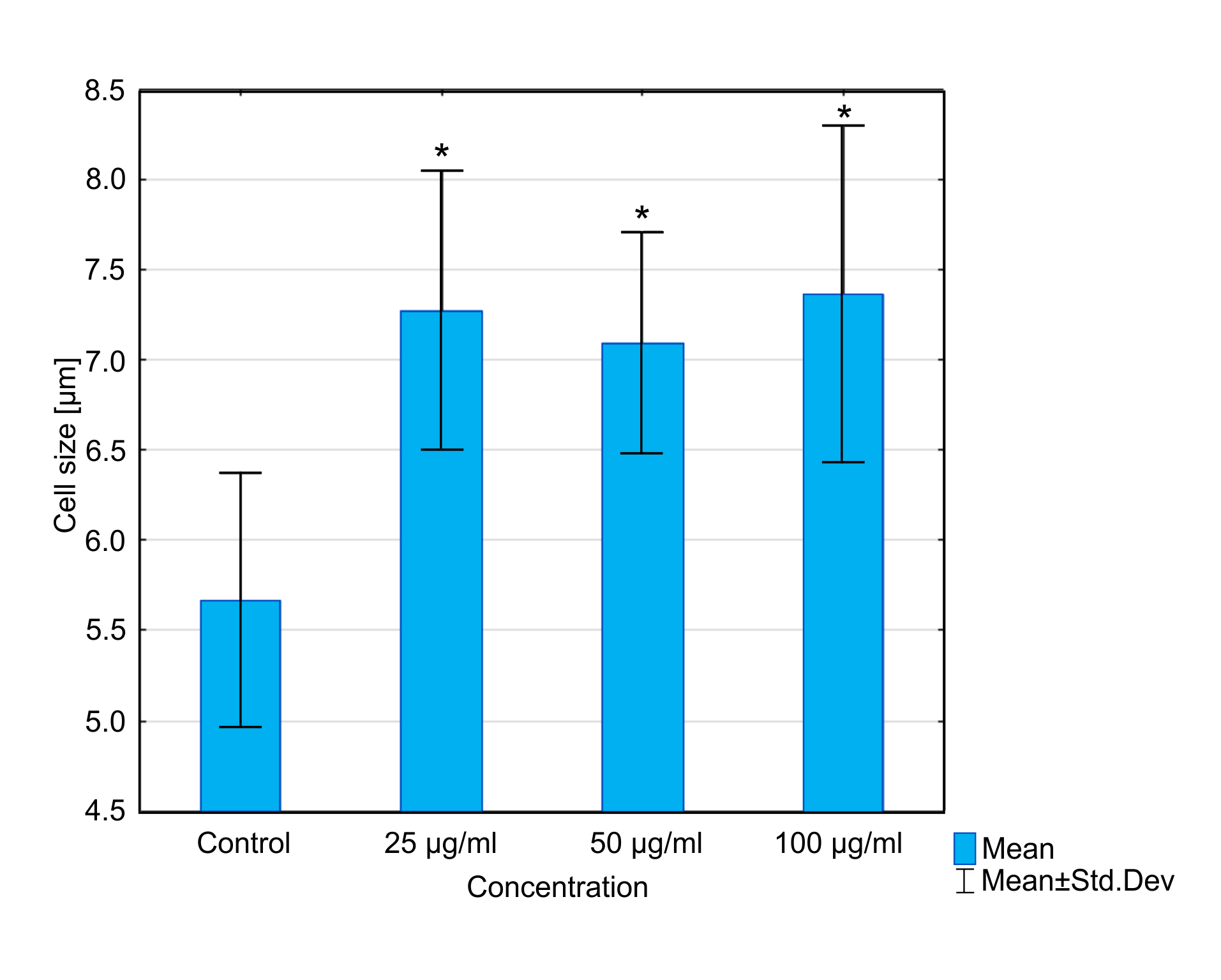

Supplement: Supplementary file 1 — Supplementary Figure S1. [file 41598_2023_41281_MOESM1_ESM.tif]

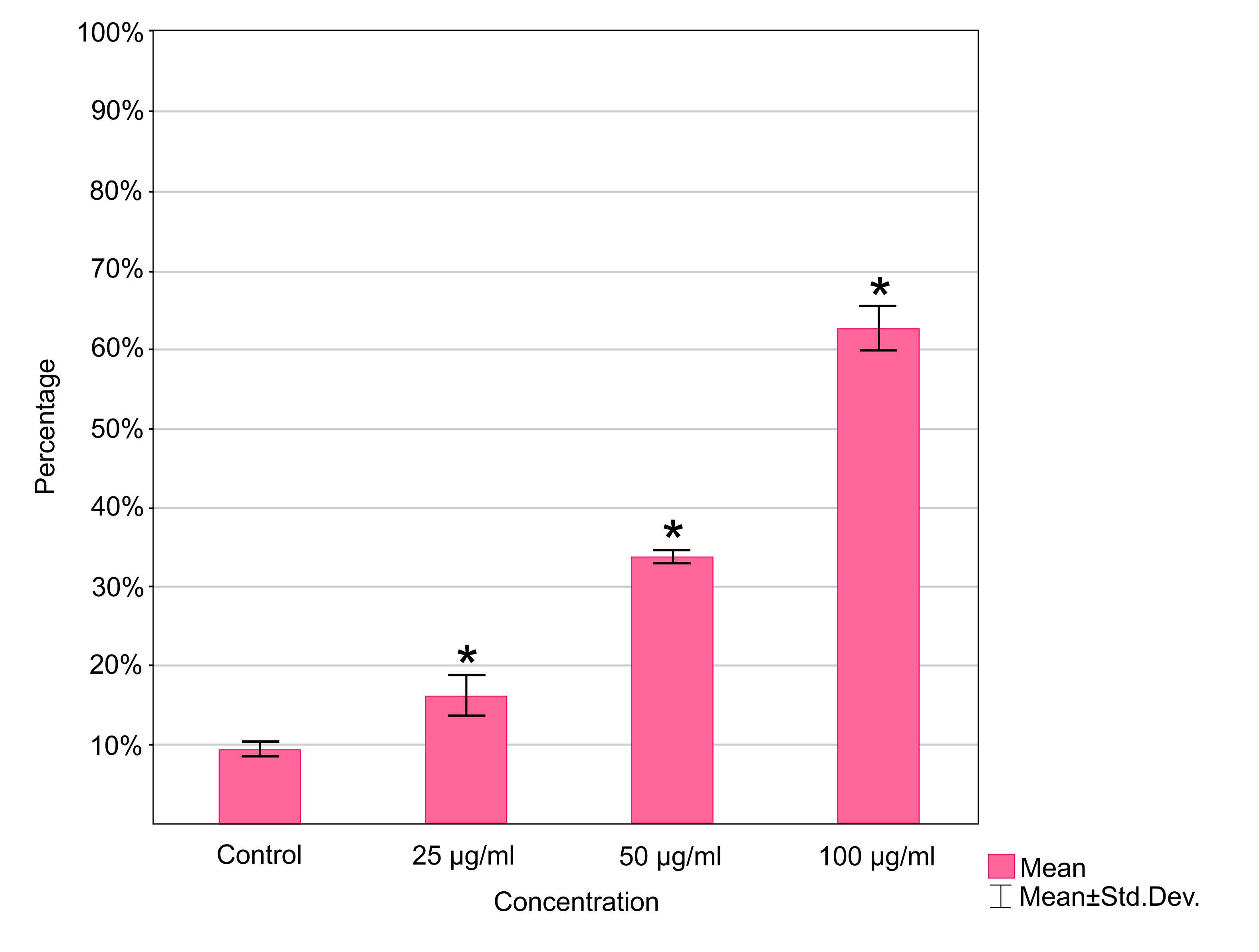

Supplement: Supplementary file 2 — Supplementary Figure S2. [file 41598_2023_41281_MOESM2_ESM.tif]
