# Supplementary material for: Autophagy of Candida albicans cells after the action of earthworm Venetin-1 nanoparticle with protease inhibitor activity
Source: Sci Rep. 2023 Aug 30;13:14228. doi: 10.1038/s41598-023-41281-4 (PMC10468520; doi:10.1038/s41598-023-41281-4)
Supplement: Supplementary file 3 — Supplementary Information. [file 41598_2023_41281_MOESM3_ESM.docx]

**Autophagy of *Candida albicans* cells after the action of earthworm Venetin-1 nanoparticle with protease inhibitor activity**

**Supplementary information**

**Fig. S1.** Average cell sizes in cultures treated with Venetin-1. Statistically significant changes were marked with (*). p<0.001.

**Fig. S2.** Average number of cells with visible autophagic bodies after OA staining. Statistically significant changes were marked with (*). p<0.001.

**Supplementary Tab. S1**. Results of basic statistical analysis performed for cell sizes after treatment with different concentrations of Venetin-1.

**Supplementary Tab. S2.** Results of basic statistical analysis for autophagic bodies in *C. albicans* cells.
