# Supplementary material for: Autophagy of Candida albicans cells after the action of earthworm Venetin-1 nanoparticle with protease inhibitor activity
Source: Sci Rep. 2023 Aug 30;13:14228. doi: 10.1038/s41598-023-41281-4 (PMC10468520; doi:10.1038/s41598-023-41281-4)
Supplement: Supplementary file 4 — Supplementary Tables. [file 41598_2023_41281_MOESM4_ESM.docx]

**Supplementary Tab. S1**. Results of basic statistical analysis performed for cell sizes after treatment with different concentrations of Venetin-1.

| Concentation  [µg/ml] | Number of cells | Mean [µm] | Std.Dev. | Shapiro-Wilk | |
| --- | --- | --- | --- | --- | --- |
|  |  |  |  | W | p |
| Control culture | 200 | 5,668820 | 0,707415 | 0,98791 | 0,08724 |
| 25 | 200 | 7,276640 | 0,774080 | 0,9492 | 0,0000 |
| 50 | 200 | 7,097445 | 0,614398 | 0,96617 | 0,0001 |
| 100 | 200 | 7,366360 | 0,931394 | 0,91935 | 0,0000 |

**Supplementary Tab. S2.** Results of basic statistical analysis for autophagic bodies in *C. albicans* cells.

| Concentration | N | Mean | Std. Dev | Shapiro-Wilk | |
| --- | --- | --- | --- | --- | --- |
|  |  |  |  | W | p |
| Control | 3 | 9,44% | 0,00963 | 0,98339 | 0,75314 |
| 25 µg/ml | 3 | 16,25% | 0,02586 | 0,9685 | 0,65922 |
| 50 µg/ml | 3 | 33,85% | 0,00821 | 0,99684 | 0,89254 |
| 100 µg/ml | 3 | 62,70% | 0,02780 | 1 | 1 |
